# Reproductive trauma, vulnerable mothers, and disenfranchised grief: reflecting on the affective dimensions of surrogacy practice in Indian literary and film narratives

**DOI:** 10.1080/26410397.2025.2477378

**Published:** 2025-03-19

**Authors:** Manali Karmakar

**Affiliations:** Senior Assistant Professor, Critical Medical Humanities, Division of English, School of Social Sciences and Languages, Vellore Institute of Technology, Kelambakkam, India.

**Keywords:** Indian film and fiction, reproductive decision-making, reproductive trauma, intending mothers

## Abstract

Drawing on an interdisciplinary approach, this study adopts and appropriates critical cultural theories such as Julia Kristeva’s abjection and Pierre Bourdieu’s social theories to examine the entangled and affective complexities of intending mothers, their reproductive trauma, and disenfranchised grief through the lens of Indian films such as *Filhaal* (2002) and works of fiction such as *Baby Makers: A Story of Indian Surrogacy* (2014) and *Kartikeya: The Destroyer’s Son* (2017). Ample research has examined surrogate mothers’ precarious position in the context of a surrogacy arrangement. However, not much has been discussed to reflect on the vulnerable status of the intending mothers who resort to surrogacy to fulfil their desire for motherhood. Thus, this study aims to highlight the significance of the selected fictional accounts to unfold the vulnerable and marginalised status of the intending mothers in a patriarchal society like India, where they find acceptance for their womanhood and earn respect and autonomy only through the power of their womb. The paper adopts generic fluidity and intersectionality as a methodology to critically analyse how the selected literature and film narratives can aid in instilling in us sensitivity towards the complex sociocultural positionality of the intending mothers who are normatively represented in popular discourses as immoral and monstrous. Emphasising the significance of the human rights-based approach to sexual and reproductive health, this research advocates for developing a non-discriminatory attitude towards intending mothers whose reproductive decision-making, privacy, and confidentiality related to the use of reproductive technology should be treated with respect and dignity.

## Plain Language Summary

There has been ample research to understand the vulnerable status of surrogate mothers. But little research examines how psychological, social, and cultural challenges affect the decision-making of intending mothers. Intending parents enter into a contract with a surrogate to bear their children. This article analyses how some Indian films and stories treat the difficulties intending mothers face. Mainstream society does not usually acknowledge any grief they may feel. This research advocates acknowledging the grief of the intending mothers. The article spotlights the intending mothers’ vulnerability, reproductive trauma, and disenfranchised grief. It aims to contribute to research narratives on sexual and reproductive health and rights (SRHR). SRHR discourse works towards a society that acknowledges reproductive decision-making as human rights.

## Résumé

Se fondant sur une approche interdisciplinaire, cette étude adopte et s’approprie des théories culturelles critiques telles que l’abjection de Julia Kristeva et les théories sociales de Pierre Bourdieu pour examiner les complexités affectives et enchevêtrées des mères d’intention, leurs traumatismes reproductifs et leur deuil non reconnu socialement à travers le prisme de films indiens tels que Filhaal (2002) et d’œuvres de fiction comme Baby Makers: A Story of Indian Surrogacy (2014) et Kartikeya: The Destroyer’s Son (2017). De nombreuses recherches ont examiné la position précaire des mères porteuses dans le contexte d’un accord de maternité de substitution. Néanmoins, peu de réflexions ont été menées sur le statut vulnérable des mères d’intention qui ont recours à la gestation pour autrui pour satisfaire leur désir d’enfant. Cette étude vise donc à mettre en évidence l’intérêt de certains récits fictifs pour dévoiler le statut vulnérable et marginalisé des mères d’intention dans une société patriarcale comme l’Inde, où elles obtiennent la reconnaissance de leur féminité et gagnent le respect et l’autonomie uniquement par le pouvoir de leur utérus. L’article adopte la fluidité générique et l’intersectionnalité comme méthodologie pour analyser de manière critique comment les récits littéraires et cinématographiques peuvent aider à nous transmettre une sensibilité à l’égard de la position socioculturelle complexe des mères d’intention qui sont représentées normativement dans les discours populaires comme immorales et monstrueuses. Soulignant l’importance de l’approche fondée sur les droits humains en matière de santé sexuelle et reproductive, cette recherche plaide pour l’adoption d’une attitude non discriminatoire à l’égard des mères d’intention dont les décisions en matière de procréation, la vie privée et la confidentialité liées à l’emploi des technologies reproductives doivent être traitées avec respect et dignité.

## Introduction

Various international conventions such as the 1994 International Conference on Population and Development (ICPD), the Convention on the Elimination of All Forms of Discrimination against Women (CEDAW), and the United Nations (UN) Sustainable Development Goals (SDGs) recognise sexual and reproductive health and rights (SRHR) as an integral part of human rights. Paragraph no 7.3 of the ICPD Programme of Action declares unequivocally that the reproductive rights of an individual or couples are premised on the freedom and entitlement to decide, free from violence, coercion, and discrimination, “whether to (not) have children, how often and when to do so, having the necessary information and means to make such decisions” (p. 60).^1^ The Government of India, as one of the signatories of international conventions, has instituted legal mandates to prevent gender-based violence to enable women to exercise their SRHR free from stigma and discriminations. The SRHR also falls within the purview of Articles 14 and 15 (the right to equality and non-discrimination), and Article 21 (the right to life) of the Indian Constitution. Furthermore, the Medical Termination of Pregnancy Act (MTPA) 2022, Surrogacy Regulation Act (2021), and Surrogacy (Regulation) Amendment Rules, 2024, are enforced by the Indian government to safeguard the sexual and reproductive choice, decision-making, and bodily integrity of women.

However, this study argues that the evolving legal and academic narratives do not devote enough space to acknowledge and cognise the complex journey of reproductive decision-making and the affective challenges of an intending mother in the context of surrogacy. Even way back in 1996, Helena Ragoné lamented that “until now the primary research focus has been on the surrogate mother herself, and that there have been no ethnographic studies on surrogate programmes and commissioning couples” (p. 353).^2^ She finds problematic the explanation offered in studies on surrogate motherhood for the decision-making of a commissioning couple. Ragoné contests the dominant narrative that a surrogacy arrangement turns out to be an easy recourse for commissioning couples to have a child who is genetically related to at least one member of the couple. She argues that assistive reproductive technologies (ART) such as surrogacy deconstruct the synchronous connectivity between woman, womb, and mother, thus problematising a couple’s normative notion of the reproductive story, i.e. that procreation is an integral part of a heteronormative relationship where the woman is biologically determined to conceive and give birth to their child. Ragoné stresses that the rupture in a couple’s reproductive story gives birth to a series of affective challenges and dilemmas that should receive critical attention in academic discourses to understand the impact of surrogacy on the lives of commissioning parents. Ragoné’s revealing of this key research gap finds resonance in the works of Teman.^3–5^ Stuvoy and Shah et al. argue that with the implementation of the Surrogacy (Regulation) Act 2021, which strictly implements altruistic surrogacy, it is high time we discuss the “other side of the surrogacy story”: the experientialities of the intending parents who occupy an equally vulnerable state to the surrogate mothers (para 1).

Assaysh-Öberg et al.^6^ bemoan the fact that although “infertility is one of the components of SRHR, [it] is not as widely addressed as pregnancy, birth, and contraception” (p. 1). Foregrounding a range of psychobiological, social, and spiritual difficulties that women undergoing infertility and infertility treatment encounter – including physical pain, loss, grief, loneliness, shame, isolation, marital conflict, and financial constraints – the researchers discuss infertile women’s precarious state. Sharma et al.^7^ reveal that in India, many women undergoing infertility treatment are also the victim of domestic violence. Sharma and Shrivastava^8,9^ draw attention to psychological distress that engulfs women during their infertility treatment. They state that although “infertility is not a mortal condition, being diagnosed as infertile can be nerve-racking experience for couples” (p. 1).^8^ Patel et al., who studied 300 women from India undergoing infertility treatment, noted that 78% of them have psychological issues, while 45% have diagnosable psychiatric problems. Shah et al. extend the discussion on infertility and infertility treatment a step further by exploring the impact of the triadic affective relationship – commissioning couples, surrogate mothers, and infertility clinic – on the intending couples. They highlight that “current surrogate research primarily focuses on commercial surrogacy with a particular emphasis on the experience of surrogate mothers; whereas the intending parents’ voices are dominated by Western perspectives” (para 1). The “experience, motivation, and decision-making of Indian parents are different and are strongly influenced by their experience of pronatalist coercive social structure” (para 3). Shah et al. also unveil the lived realities of many intending parents as they undergo reproductive oppression in the form of stigma and verbal abuse. They assert that in the narrative of surrogacy in research, the Indian intending parents’ voices are “only a whisper” (para 1). Agreeing entirely with Shah et al.’s arguments, this study analyses how the intending mother’s emotional integrity further gets problematised in the journey of surrogacy where the couple takes a “woman’s womb on rent” to make up for their reproductive lack.^10^

The lack of judicious representation of the affective complexities of intending parents is also underscored in the work of Teman who highlights how misrepresentation of intending mothers may further jeopardise the goal of accomplishing reproductive justice for all. She discusses how media representation carries a “tone of accusation” against women who choose non-normative reproductive measures. Teman states that in popular accounts, surrogacy is dramatised as an easy option for elite women to avoid “stretch marks”. Teman’s portrayal of elite women is also reinforced in Indian popular cultural narratives like the Hindi animated series on YouTube, *Chat Pati Kahaniya*^11^ (spiced-up interesting stories) that dramatises surrogacy as an easy recourse for women who are figure-conscious and would like to save themselves from birth pain. Similarly, Bollywood films like *Welcome Obama* (2013), *Badnaam Gali* (2019), *Mimi* (2021), *Yashoda* (2022), and *Dukaan* (2024) have continued to present a lopsided image of the intending parents. This form of half-baked representations should be consumed with caution. For instance, the film *Dukaan* overarchingly represents the intending mother as immoral and excessively moneyed, thereby dramatising her motherhood under a gaze of suspicion and unreliability. The film peripherally captures the “social death” experienced by Diya and Armaan (the intending parents).

Capo and Lazzari^12^ argue that weeding out such wrongful representations from our cultural psychology is central to reproductive justice. Storytelling forms a crucial feature of reproductive justice theory because it allows us to resist and reclaim the unheard voices that have been ignored by dominant cultural narratives. “Fiction, nonfiction, and film/television/YouTube do more than document contemporary concern” (p. 5).^12^ The style of representations influences our thought process, thereby creating possibilities to create constructive ideologies. Hence reproductive storytelling is a form of activism. Representation of reproductive phenomenology circulates, persuades, augments, and energises the social justice movement. Mendes^13–15^ argue in a similar vein. Mendes states that it is important to analyse to what extent “the production and circulation of knowledge about assisted reproductive technology in India is widening the spectrum of representations to capture the voices” and sociocultural positionality of different stakeholders like the surrogate mothers, the surrogate’s family members, the medical clinical personnel, and the commissioning parents (p. 79).^13^

Acknowledging the significance of literary and film narratives, this study aims to bring to light the voices of intending mothers through a close reading of Meghna Gulzar’s film *Filhaal* (2002),^16^ Gita Aravamudan’s story *Baby Makers: A Story of Indian Surrogacy* (2014), and Anuja Chandramouli’s mythological fiction *Kartikeya: The Destroyers Son* (2017). The underlying reasons for the emotional complexities buried deep down in intending mothers manifesting as guilt, anger, and frustration in films like *Badnaam Gali*, *Yashoda, Mimi*, *Welcome Obama*, and *Dukaan* can be gleaned from the narratives of *Kartikeya*, *Baby Makers*, and *Filhaal*. Macaskill^17,18^ argues that receiving support at the time of grief is a human right. Therefore, Macaskill urges considering bereavement support as an integral component of human rights, as this acknowledgement enables a bereaved person to ascribe meaning to the loss and to articulate their grief and mourn the loss without the fear of being stigmatised and mocked at by society. The argument finds strong approval in the works of Gray and Lassance^19^ and clinical psychologists Jaffe and Diamond^20^ who endeavour to recognise the trauma associated with reproductive loss in the context of “non-normative reproductive phenomenology” such as miscarriage, stillbirth, infertility, abortion, and issues where a person’s reproductive dreams are precluded by life situations. Non-normative reproductive phenomenology denotes the reproductive experiences that deviate from the essentialised notion of birth-giving, i.e. that every woman is biologically determined to conceive and give birth to a baby following the normative course of pregnancy. Gray and Lassance argue that grief associated with non-normative reproductive phenomenology “is often minimized, denied, and considered to be outside the normal ‘grieving rules’ of society. Yet individuals who have suffered these losses can experience profound grief and emotional pain. Their grief needs to be acknowledged not only by themselves but by others as well” (p. vii).^19^

Gray and Lassance foreground the integral connectivity between reproductive loss and trauma, stating that “the loss of the assumptive world, the loss of the expectation of a perfect pregnancy, and the loss of a worry-free, innocent, and joyful subsequent pregnancy” (p. 83) transform an individual or a couple into traumatised and grief-stricken humans for whom being emotionally shattered, fearful, dissected, and angry becomes a state of being in the world.^19^ The trauma of reproductive loss may be experienced both at the embodied and symbolic levels. Diamond and Diamond explain, “Fundamentally, reproductive trauma derives from the disruption to a person’s parental identity, or ‘reproductive story,’ the unconscious narrative that people develop from childhood, when ideas of what it will be like to be a parent begin” (p. 3).^21^

Therefore, the study of reproductive trauma, in our opinion, is crucial to understanding how the triad of affective relationality between the intending mother, surrogate, and the baby has given birth to new orders of emotional complexities and challenges for the intending mothers.^20,22^ Kothari and Sriram mention that in a pronatalist society like India, where procreation is intrinsically valued to continue the genetic lineage, “women’s fertility is conceived to be of key importance to all family members and society” (p. 212).^23^ They argue that in a patriarchal family, “freedom comes for women after the birth of a child, more specifically a male child” (p. 213).^23^ Aravamudan says, “Motherhood is a state of being, which has, from time immemorial, been defined by a set of cliched, internalized words that are as powerful as they are evocative” (p. 1).^22^ Drawing on the concept of social suffering as explained by Kleinman, Das, and Lock, it may be argued that insidious internalisation of the hegemonic discourse of motherhood by Indian women acts as a “soft knife procedure … that ruins the collective and intersubjective connections of experience and gravely damages the subjectivity” (Introduction x).^24^ In this scenario, it becomes crucial to examine how the fragmented notion of motherhood represented by surrogacy affect the subjective experientialities of the intending mother.

This study starts by seeking to examine how the fictional narratives in *Kartikeya*, *Baby Makers*, and *Filhaal* dramatise the entangled and affective complexities of surrogacy, reproductive trauma, vulnerable bodies, and disenfranchised grief. The article underscores how these selected fictional narratives also provide an insight into the complex reproductive decision-making journey of intending mothers, that may include violence, discrimination, prejudices, guilt, and abjection. A study of this kind will help us to bring forth the pain, trauma, fear, and anxiety of the intending mothers; this is crucial to enable society to acknowledge their reproductive trauma. Second, it elaborates how these selected fictional narratives will enable us to contest the dominant image of intending mothers as heartless, rich monsters who exploit surrogate mothers to fulfil their reproductive desires. Third, a theoretical framework is designed, encompassing critical theories of affect with a specific focus on Kristeva’s concept of abjection and Bourdieu’s^25^ social theory, to foreground the affective complexities and vulnerabilities that affect the subjectivity of intending mothers in a surrogacy arrangement.

### Methodology

Pierre Bourdieu^25^ explains that the perspectives, thoughts, choices, and actions of humans are complexly structured by the enmeshing relationship between “habitus”, “field”, and “capital” (cited in^26^, p. 33). Bourdieu defines habitus as a “chronologically ordered series of structuring determinations […]” (p. 86). To further explain the concept of habitus, he draws on concepts like “disposition”, “way of being”, and “a habitual state” to demonstrate how individuals’ interpretation of his their social world and their choice and decision-making are simultaneously structured by their past experiences, cultures, and values inculcated over time and the “social field” in which they are embedded (p. 214). Thus, Bourdieu defines habitus as a “structured, structuring structure” (p. 77). He posits that habitus and capital complement each other. Capital is defined as resources – cultural, social, and economic – that an individual accumulates over a period of time through his/her belongingness to a “social field” (p. 70). Cultural capital, Bourdieu argues, gets manifested in three forms: “in the embodied state, i.e. in the long-lasting dispositions of the mind and the body; in the objective state in the form of cultural goods, and in the institutionalized states” (p. 243).^27^ Bourdieu goes on to establish that “Cultural capital can be acquired, to a varying extent, depending on the period, the society, and the social class, in the absence of any deliberate inculcation, and therefore quite unconsciously” (p. 244).^27^ The concept of cultural capital is also associated with symbolic capital: how we define and identify ourselves in society. Cultural capital in its objectified state remains in the form of cultural goods and artefacts. Academic qualification is an apt example to explain the institutionalised state of cultural capital. Bourdieu describes “social capital as the aggregate of the actual or potential resources which are linked to possession of a durable network of more or less institutionalized relationships of mutual acquaintance and recognition – or in other words, to membership in a group – which provides each of its members with the backing of the collectively owned capital, a ‘credential’, which entitles them to credit, in the various senses of the word” (p. 247).^27^

**Figure 1.**
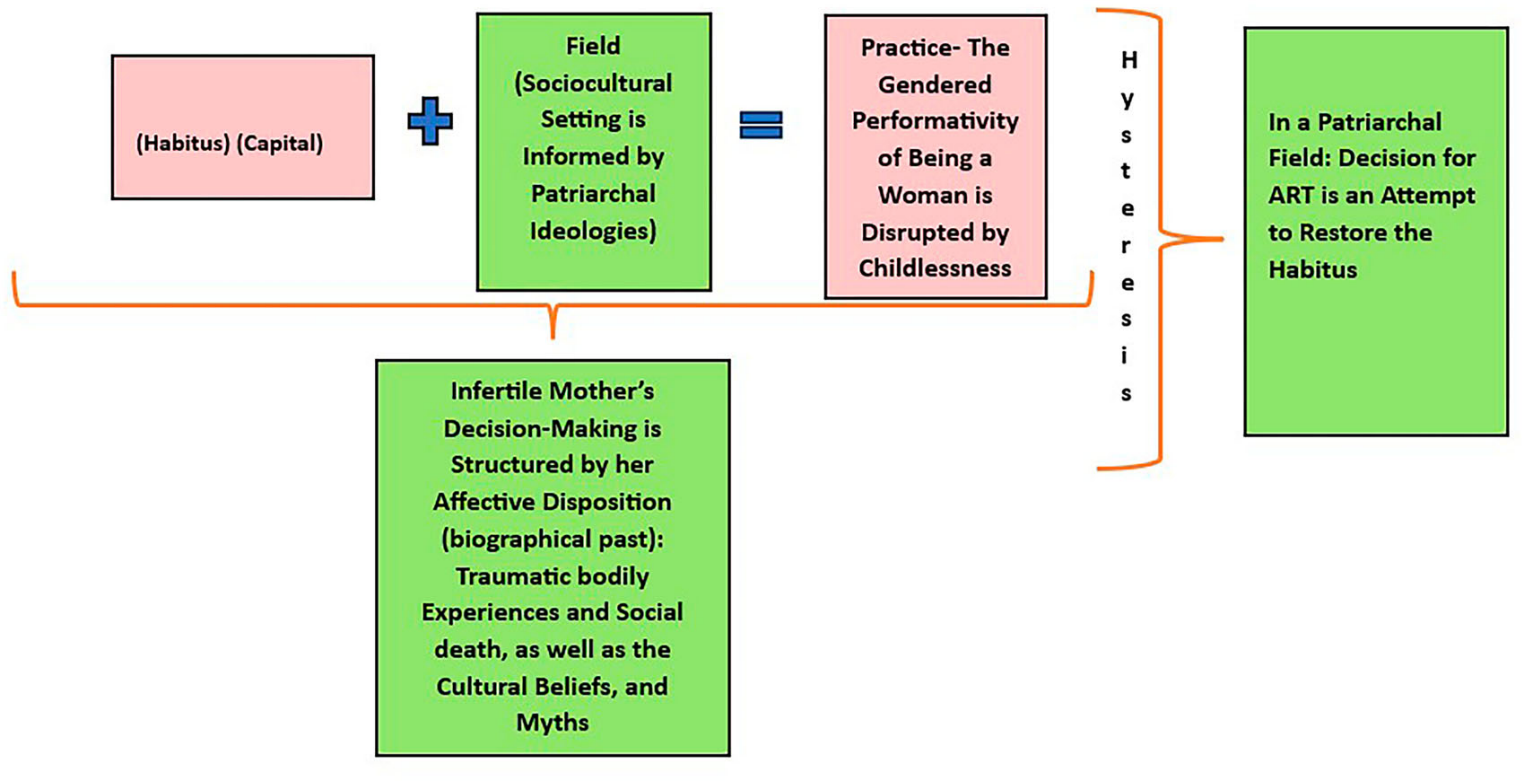
Adoption and Appropriation of Bourdieu's Social Theory Model.

The female protagonists – Parvati in *Kartikeya*, Meena, Aparna, and Sharada in *Baby Makers*, and Rewa in the film *Filhaal* – are represented as cultured women, educated and belonging to the upper class. Viewing from Bourdieu’s framework, they are endowed with cultural capital because of their birth in upper-class families, exposure to education, and entitlement to financial resources. However, when infertility emerges as a lack, it jeopardises the women’s entitlement to social capital such as motherhood. Through a close reading of the selected texts, this research foregrounds how infertility and infertility treatment problematise the mothers’ affective disposition, and their habitual state of being, thereby creating an order of unrest and anxiety that concords with Bourdieu’s explanation of hysteresis. “Hysteresis occurs where habitus falls out of alignment with the field in which it operates, experienced as lag or disconnect amid changing circumstances, where taken-for-granted assumptions seem less relevant (cited in Graham,^29^ p. 451). “It feels like living in a different time, carrying risks and opportunities”,^30^^,^^31^ (cited in Graham,^29^ p. 451). In this context, the decision-making of the intending mother to avail surrogacy turns into an effective mode to re-establish her habitus and to assert her social capital. However, Kristeva’s abjection also showcases how the journey of surrogacy may evoke both excitement and abjection within the intending mother. Below a figure is presented to interpret the habitus of the intending mother whose habitual state is shaped by the cultural psychology of patriarchy (Figure 1).

Kristeva uses the concept of “abjection” to explain the issue of anger, insecurity, and disgust that intending mothers may experience in the journey of begetting a child through surrogacy (p. 125).^32^ Kristeva explains abjection thus: “It is there, very close, but unassimilable. It solicits, disturbs, fascinates desire, which, nevertheless, does not let itself be seduced. Fearful, it turns away. Sickened, it rejects” (p. 127).^32^ Abjection may be construed as an intensive affective force that resists any form of easy definition. It may be explained as a noncodified, interactable, non-representational, asignifying order of experientialities that evolve as a result of a heterogenous affective arrangement between “an array of persons, things, artifacts, spaces, discourses, behaviours, expressions, and other materials” that have the potentiality of “affecting and being affected” by each other’s presence (p. 19).^33^ Abjection thus can be treated as an ambiguous affective state. The notion of abjection, as explained by Kristeva, challenges the labelling as “monstrous” of the intending mother who, in a traumatic state, may ask for the pregnancy to be aborted or deny the baby born with some form of disability.

**Figure 2.**
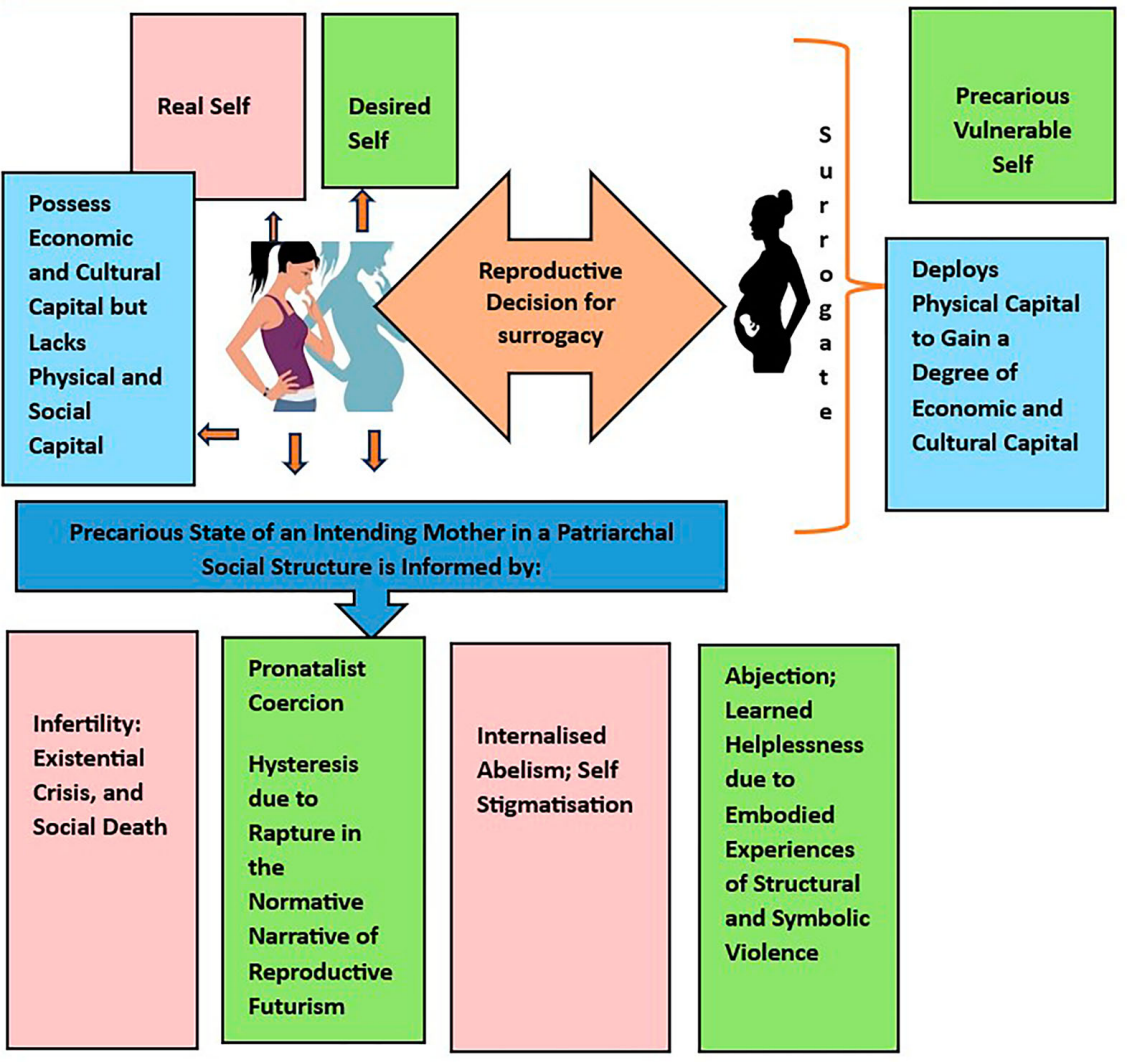
Surrogacy – Intersectionality and Interplay of Vulnerable Bodies. *Note:* The figure offers a quick overview of multiple socio-cultural and affective factors that influence the reproductive decision-making of an intending mother.

An intersectional approach is employed to demonstrate how the intending mother’s decision to restore her habitus by accepting surrogacy as a means to achieve a degree of social capital further instils a sense of vulnerability and precariousness in her. Kimberlé Crenshaw coined the term “intersectionality” in 1989 to throw light on the entangled and enmeshing nature of oppression promoted by conventional notions related to gender, race, and other relatable characteristics. She argues that oppression is neither a singular nor a binarist political process; rather, it emerges from multiple sectors that problematise the sociocultural growth and mental well-being of marginalised people. Pande also emphasised that “a meaningful transnational feminist engagement around issues like surrogacy needs to be attentive to the politics of differential locations, and the relevance of intersectionality in the experience of surrogacy” (p. 9).^34^ She stressed the importance of geopolitical locations and sociocultural context in shaping a woman’s reproductive choices and decisions. Pande’s study primarily uses the method of intersectionality to study the phenomenology of the surrogate mother. Instead, intersectionality is used here to study the geopolitical and sociocultural location of the intending parents. Drawing on concepts such as “structural violence”^35^ and “symbolic violence”,^36^ gender-based violence embedded in the pronatalist social structure is also analysed. Lee defines structural violence as “avoidable limitations that society places on groups of people that constrain them from meeting their basic needs and achieving the quality of life that would otherwise be possible. These limitations, which can be political, economic, religious, cultural, or legal in nature, usually originate in institutions that exercise power over particular subjects” (p. 127)^35^ whereas symbolic violence refers to the internalisation and acceptance of inequalities and injustices as the norms of life. Figure 2 offers a pictorial representation of the web of intersectional issues – pronatalist coercion and reproductive futurism, infertility, and associated gender violence (structural and symbolic) – that influence the intending mothers’ notions of reproductive choice and justice to achieve a degree of social capital in a societal structure informed by patriarchal ideologies. Edelman (2004)^37^ explains that pronatalist coercion and reproductive futurism are aligned to a political ideology, a social system that valorises reproductive sexuality.

Multimodal transcription, developed by Thibault (2000)^38^ for an effective analysis of audio-visual texts, is also used as methodology in this study. “The method involves breaking down a film into single frames/shots/phases, and analysing all the semiotic modalities operating in each frame/shot/phase” (p. 191).^39^ This methodology is also used for “formulating strategies for subtitling particularly in relation to the translation” of the verbal elements of the text (p. 191). The film *Filhaal* selected for the study is produced in Hindi language and hence multimodal transcription was used to transcribe significant scenes into English for the purpose of the study.

### Rationale for the choice of the texts

Birth and the preservation of lineage through non-normative reproductive methods are a recurrent theme of mythological fiction and therefore the discussion begins with the Indian cultural narratives on surrogacy, reproductive trauma, vulnerable bodies, and disenfranchised grief by discussing Chandramouli’s mythological fiction *Kartikeya*, which dramatises the birth event of Kartikeya, the son of Shiva and Parvati. In Hindu mythology, Parvati, the divine mother, is the archetypal image of womanhood and an all-powerful creative goddess of fertility. However, it is often believed that Goddess Parvati is cursed by Rati who considers Shiva and Parvati to be responsible for the death of her husband Kamadeva. Hence, out of anger, Rati curses the couple, stating that Parvati will also experience the similar grief of losing someone who is very dear to her. Rati’s curse turns true when the Goddess, due to unforeseeable circumstances, fails to conceive Shiva’s *bija* (seed/sperm) in her womb. At this juncture, Parvati’s sister Ganga comes forward to bear the child in her womb. Thus, Shiva’s seed was implanted in Ganga’s womb, turning her into Kartikeya’s “surrogate mother”.^40^ Hence, “The birth of Kartikeya also known as Subramanya Swamy is explained as another example of the art of surrogacy that existed during ancient India”.^9,40^

What makes Chandramouli’s *Kartikeya* an important mythological fiction, to begin the analysis of non-normative reproductive phenomenology, is that it draws on the birth event of Kartikeya to dramatise the reproductive trauma of Parvati. The article argues that Parvati’s grief alludes to the reproductive trauma of twenty-first-century intending mothers. Devdutt Pattanaik,^41^ a storyteller and mythology expert, argues that myths ≠ *mithya* (false statements), but are instead “subjective truths”, a culture’s truths that have a deep impact on our cultural memory and insidiously shape the cultural psychology of contemporary society. Myths are symbolic capital. Myths guide our morality and value system, thereby allowing the internalisation of certain orders of truth to live our life as social beings. They structure our habitus. The intertextual connection among the multiple genres – mythological fiction, fiction, and film – chosen for this study, turns into an apt tool to make a comparative analysis of how the habitus of modern India’s intending mothers, as captured in *Baby Makers* and *Filhaal*, is influenced by the reproductive grief and trauma of Goddess Parvati. The theme of reproductive grief and trauma of the intending mother finds resonance in all the selected texts, and hence thematic narrative analysis and generic fluidity are used as methodology for analysis. Riessman^42^ defines thematic narrative analysis as a research methodology where the theme is the prime focus. Nancy defines generic fluidity as a strategy where “a text is a deliberate assemblage of thematic messages – rendered in overlapping authorial voices, combining narrative, poetry, essay, polemic and more – and is thus an example of queered multivocal writing” (p. 7).^43^ Generic fluidity aligns with Kristeva’s notion of intertextuality that considers literary text as a “dynamic site”, which serves as “an intersection of textual surfaces … as a dialogue among several writings” (p. 65).^44^

The selected texts are thematically arranged to support the analysis of the reproductive trauma. *Kartikeya*, through the lens of Parvati, initiates the discussion. *Baby Makers* deals with issues of reproductive trauma by dramatising the intersectional relationship between reproductive oppression, guilt, and fear associated with the decision-making of availing surrogacy, and *Filhaal* explains the root cause of abhorrence and disgust that intending mothers may develop for the surrogate mothers in their journey of begetting a child through a surrogacy arrangement. The female protagonists, dramatised in the selected texts, represent the vulnerable position of the upper-class empowered Indian intending mothers. Their subjectivity corroborates the concept of “vulnerable empowered women” described by Tasha N. Dubriwny.^45^ The concept of vulnerable empowered women foregrounds that although in “post-feminist and neoliberal healthcare narratives” educated women are projected as empowered decision-makers, “the choice is yours” narrative is informed by the hegemonic discourses of the patriarchal consumer culture (p. 8). Dubriwny’s concept aptly captures the decision-making dynamic as represented in *Baby Makers* and in *Filhaal*. Similar to Parvati, the female protagonists are biologically incapable of conceiving their child, and hence they are compelled to choose surrogacy to fulfil their reproductive desires. Surrogacy is represented as a secret affair in all the selected texts. The fear runs across these texts that if it is disclosed, it will bring to light not only their biological incapability but also their decision-making, and the child born out of assisted reproductive technology (ART) will be stigmatised and mocked by society, runs across these texts. These texts, dating from 2002 to 2017, aptly capture the reproductive trauma related to surrogacy that continues to haunt the decision-making of Indian couples. The fictional dramatisation of intending mothers’ trauma find resonance in a recently published article in the *New York Times* that argues that in India, ART carries “widespread stigma including rumours that babies born through the therapy are made in ‘plastic boxes’ or that such children are someone else’s genetically” (para 13).^46^

### Analysis and discussion of the selected texts

#### Chandramouli’s *Kartikeya*

The mythological fiction begins with the narration of the birth event of Kartikeya, for which the *Devatas* (a group of deities who promotes the practice of Vedic religion) have been waiting for aeons. According to the divine prophecy, the son of Shiva and Parvati will release the *Devatas* from the aeons of grief and misery that have fallen upon them after a demon, Soorapadma, dethroned Indra, the king of Heaven. Contrary to this, for Parvati, the baby is not a war machine. The birth of Kartikeya will be the manifestation of her love for Shiva. Parvati’s heart’s desire to experience the incredible happiness of conceiving Kartikeya is shattered when the *Devatas* sneak into her abode while she was propitiating Shiva in the anticipation of getting impregnated by the fruit of his loins. In their compromising state, when Parvati feels the presence of the *Devatas*, she

*“pulled herself free, wrenching apart from her lover, experiencing a feeling of loss so profound, the tears spilled freely from her eyes, blinding her as she retreated into the dark interiors of the cave … At the moment Shiva spilled his seed”* (p. 22).^47^

Agni (the god of fire), one of the Devatas who sneaked into the cave, acts on reflex, gathers the seed in his palms, and along with Vayu disappears in the distant skies. Parvati feels humiliated by the presence of the outsiders; but the sense of humiliation morphs into grief, anger, and despair as the reality gradually sinks down into her. Parvati has lost one of her life’s opportunities – to bear her child in her womb.

The feeling of the empty womb crushed down upon Parvati; whereas Shiva seems to be calm. Shiva exhibits neither anger nor regret for not being able to reprimand the Devatas for their audacity. Parvati is more devasted when Shiva says that what has happened is destined and hence there is nothing to mourn. Shiva in a measured tone says

*“My seed had been charged by the power of penance, and if I had discharged it into your womb, the power would have been neutralized which would not really suit the purpose of those who have need of the Destroyer’s son to vanquish their enemies now”* (p. 25).

For the gods and goddess, Parvati is the divine mother, *prakriti* (nature), and the goddess of fertility, whereas Shiva denies her the “right” to conceive their son in her womb because he thinks that her womb is too weak to carry their son (p. 23). Shiva’s disregard for Parvati’s reproductive ability and his flattened emotional response to her grief further accentuates Parvati’s trauma. Shiva’s statement reminds Parvati of Rati’s curse: “I hope something terrible happens to them! It is evil of me, but I genuinely want Parvati to know what it feels like to lose something dear to her!” Parvati’s “chest heaved with the vehemence of her grief and rage”, as she silently listens to Shiva’s defence of the act of the *Devatas* (p. 24). The divine mother “clutched her womb, and her cry was that of a wounded animal” (p. 23). Her whole body fumed with anger and abhorrence. In a fiery voice, she curses the *Devatas*: “You have taken something that was *mine by right* [my emphasis],” she began, “[…] Mark my words, my child will be restored to me, for I am blameless. As for your wives, their wombs will remain arid and barren forever” (p. 23). It may be argued that Parvati, by cursing the *Devatas*, demands the community to share the grief of her reproductive crisis. Because it is only when the *Devatas* experience the trauma, they will understand why a woman mourns when she loses a chance to bear a child in her womb. Thus, the community will be able to acknowledge the pain of her reproductive loss.

Parvati firmly states that no power on earth can shatter her maternal-fetal bond with Kartikeya. She emphasises “he is mine. My own son! Nobody can succeed in taking him away from me anymore than those who would seek to separate us” (p. 25). She pledges to get her son back and to raise him with her own hand. The birth of Kartikeya stands as an example of traditional surrogacy. In the context of Kartikeya’s birth, Shiva’s seed is implanted in Ganga’s womb, hence turning her into the biological and birthing mother of Kartikeya. Unlike Shiva, Ganga acknowledges Parvati’s grief. She explains how the rupture in the normative reproductive narrative of Parvati leaves the Goddess traumatised:

*“There she was moaning and groaning, whining about having lost the chance to carry Shiva’s seed in her womb, having no doubt, that her belly would grow fat on his distillate and dreamed about delivering his child while he held her hand like a dutiful husband and householder”* (p. 29).

When it’s her turn to depart from the baby, Ganga too admits that Parvati’s experience is excruciatingly painful because it is not easy to accept the fact that “something one cared for more deeply than anything else [could be] cruelly wrenched away from her” (p. 29).

### Aravamudan’s *Baby Makers*

*Baby Makers* employs a third-person narrative style to tell the life stories of the intending couples – Meena and Ram, Sharada and Rajappa, and Aparna and Venky. Married for 10 years, Meena and Ram work for companies based in the United Kingdom and hold good positions with high salaries. In the course of their marriage, Meena has experienced multiple miscarriages and has undergone multiple rounds of infertility treatment. A slightly older couple, Sharada and Rajappa, married for 20 years, face the same issue as Meena. After three miscarriages, the doctor states that Sharada’s womb is too weak to carry a baby and suggests that the couple “find a nice healthy young woman” to give birth to their baby (p. 11).^19^ The couple hire Manju to fulfil their reproductive desire and to save themselves from being ostracised by society. Like Meena, Aparna is a software engineer who also has experienced three miscarriages and has undergone a series of infertility treatments. Despite all efforts, Aparna fails to conceive. The couple avails for surrogacy using their genetic material. However, they fail there as well, leaving Aparna devastated. However, Aparna’s reproductive desire does not die. Thus, Aparna and Venky undergo IVF treatment with Venky’s sperm and a donor egg that is implanted in the surrogate Deepa, who later gives birth to a baby. The reproductive trauma of the couples, as dramatised in *Baby Makers*, foregrounds how the couples make varied attempts through technological interventions to battle through the phase of hysteresis and to reterritorialise their habitus.

Journalist Virani^48^ explains how difficult it may be for a woman to make a decision for surrogacy. Many times, a woman may not be willing to accept surrogacy as a solution because “somewhere deep within her, she knows why; even as its birth mother this will not be completely her baby. It will be his and it will be his family’s, to keep their maan–maryada [honour] in society. She does not want to be just an honour-birth enabler; but she will not, how can she, say such words” (p. 35).^48^

To regain their lost social capital, women such as Meena and Sharadha decide to venture into the journey of surrogacy because in our society children are an extension of our married life. Virani explains: “They try, they cannot. Infertility. They try non-complex acts of assisted reproduction. They decide to try again. Because technology is available” (p. 31). Virani’s explanation underlines the paradox in the narrative of the freedom of choice and decision-making in the current culture of assisted reproduction. She argues that the technologies that were once introduced to make women’s reproductive choices flexible are now further constraining women’s choices by “reinforcing the oppressive circumstances in which childbearing decisions are made” (p. 31).^48^ The medical narratives make false claims that in ART there is nothing to lose. Gone are those old days when we needed to give social recognition to the woman whom the husbands used to marry because the first wife was not able to bear the lineage. Hence many women, as portrayed through fictional characters like Meena, Sharada, and Rewa, endeavour to have a family through surrogacy. Although all the female protagonists in *Baby Makers* have undergone acute pain and trauma in their baby-making journey, Virani’s explanation of reproductive oppression is aptly captured in the narratives of Meena. Meena’s reproductive trauma throws light on how the intersectional existence of structural and symbolic violence in the form of pronatalist social structure, reproductive coercion, and internalised ableism informs the reproductive decision-making of an intending mother. Meena recalls: “The four years of expensive and painful infertility treatment; the long tension-filled months when nothing seemed to work; the tears, the trauma. And then the decision that had suddenly brought some light into their lives” (p. 31).^19^ Meena recollects how her in-laws never acknowledge the trauma and pain she has been undergoing due to recurrent miscarriage and infertility treatment. Listening to taunts and blame by her in-laws had become a daily occurrence. Recurrently, her in-laws remind her that a woman’s “place is in the house. Her first duty is to reproduce” (p. 32).^19^ Her infertility, the failed infertility treatment, and the in-laws’ insults lead Meena to believe that she is responsible for her husband’s unhappiness. In due course, Meena develops self-stigmatisation and begins justifying her mother-in-law’s abuses, thinking that she deserves to be blamed. Like her in-laws, she starts believing that she should do everything to give her husband a child. These episodes of reproductive oppression aptly demonstrate how the structural violence embedded in the pronatalist social structure gradually gives birth to symbolic violence in the forms of self-blame, internalised ableism, and self-stigmatisation.

The surrogacy journey is complexly enmeshed with ambiguous emotions – guilt, anxiety, shame, jealousy, excitement, and amusement – that resist any form of easy categorisation. Sharada explains that when she first steps into a surrogate house to finalise the woman who will be carrying her child, the surrogate house engulfs her with “strange kind of emotions” (p. 63).^19^ Sharada wonders:

*“How easily had these women become pregnant! And she had been trying for years now. Why was nature so unfair? […] Maybe they were doing this for the money. And here she had everything money could buy, but no baby*” (p. 68).

Sharada and Meena keep the surrogacy process a secret. They do not want to experience the inquisitive gaze of society any more. There is no baby shower and no ceremony to reveal to the world that they will also feel the happiness of being a mother soon. Sharada reflects, “But, this time, she had to do it very quietly because she obviously couldn’t invite her relatives to a ceremony for her surrogate” (p. 138). The experience of alienation is also accompanied by the feeling of guilt, which is confirmed by Jhutty^49^ as an integral part of surrogacy.

The experience of guilt varies for Sharada and Meena. Sharada feels guilty that because of her inability, another woman has to undergo the trouble of IVF. Meena’s guilt-ridden conscience emerges from multitudinous factors that she has to navigate and negotiate with to shut the judgemental gaze of society. Because of her, “their [her in-laws’] wonderful family line wouldn’t be carried forward because their only son had no children” (p. 33),^19^ which gives rise to the feeling of guilt. Secondly, Meena’s remorse emanates from the lies that she has to tell her in-laws, friends, and relatives to conceal the fact that she has hired a surrogate to experience motherhood. Thirdly, like Sharada, she regrets that Alice has to undergo so much pain to help her experience the happiness of being a mother. Meena silently contemplates: “Alice would have had to take hormone treatment. How had that affected her? And all the shots” (p. 78). Finally, Meena suffers pangs of guilt when she meets her friend Shirin, who teaches gender studies at a college. When Meena shares her news of hiring a surrogate, a shocked Shirin asks, “What, Meena! You of all people! How could you even think of using another woman’s body to have your child? Why? Why? If you were so desperate you could have adopted” (p. 124). Meena’s defence fails to convince Shirin. Meena argues that Alice has consented to the process voluntarily and it is just like any other business or profession we do. Alice

*“is selling her services just as a domestic worker, a software engineer, or a doctor. As if I would exploit another woman. I am paying her for the service she is rendering me. And that money is going to be very useful to her. She told me so herself*” (p. 125).

Meena’s arguments boomerang on her own self. Already embarrassed, her feelings reach a threshold when Shirin says,

*“You are an intelligent, educated woman, Meena. Surely, you know that you don’t have to prove your womanhood by producing a genetically related child. Your fertility, or lack of it, cannot define you”* (p. 125).

In a reflex reaction to the conversation, Meena tries to find out more about negative aspects of surrogacy, and what she learns by browsing the internet makes her feel ashamed of her decision. She feels bad that she has brainwashed herself into thinking of the woman’s womb as just a container. Alice is not just a womb; she is more than her womb. Alice is a person as well, but that does not change the fact that Meena cannot publicly accept the surrogate’s role in producing her babies. Acknowledging Alice as her baby-birthing mother may jeopardise her credentials to motherhood as a social capital. Thus, Meena comes to the conclusion that at present she has to stay blind to all the ethics and focus on her own good and well-being. To restore her habitus, she needs the babies. Meena collectively mourns the situation of the modern vulnerable woman thus:

*“[…] eyes filled with tears. There it was again, the importance of a functional womb. All her education, all the gold medals she had won for her academic prowess. Everything had come to naught because of her inability to reproduce”* (p. 78).

The episode beautifully demonstrates the importance of physical capital in a pronatalist coercive social structure. Physical capital denotes “the development of bodies in ways recognized as possessing value in social fields, and the conversion of physical capital as involving the translation of bodily participation in work, education and other fields into other resources” (p. 474).^50^

Shilling argues that physical capital, if rightly used, may help a person to achieve economic, cultural, and social capital. Thus, it may be argued that since Meena lacks physical capital, her “damaged womb” invalidates the importance of her cultural capital and her entitlement to social capital.

The trauma of using a fake belly aggravates her pain. If she has to convince her in-laws about her pregnancy, then she has to look and act like a pregnant woman. Radha, Meena’s friend and a doctor, asks her to get herself mentally prepared for faking the pregnancy. Alluding to many couples, Radha says that using fake bellies becomes a requirement for many women to safeguard themselves from shame and societal stigma. The episode corroborates the real-life issues of women opting for strap-on bellies to hide their decision of availing surrogacy. In a newspaper article, assistant editor Radha Sharma of *The Times of India*, Ahmedabad, reports that many women who are doctors and graduates from institutions of repute such as the Indian Institute of Technology (IIT) opt for a “strap-on stomach” because in traditional communities they fear to disclose to their in-laws and relatives that they are biologically not capable of bearing the child and therefore have appointed a surrogate to fulfil their dream of motherhood.^51^ In *Baby Makers*, in one of the deciding moments, Meena decides to disclose the truth about surrogacy to her in-laws. She gets mentally ready to bear the consequences.

After the birth of the twins, the news is curated by Meena’s husband Ram so that his parents should accept the daughters. Meena is apprehensive that her in-laws may not accept the babies. Firstly, because they are born out of surrogacy, and secondly, they are girls. Contrary to her fear, the babies are happily accepted when Ram specifically mentions: “Darling, beautiful little girls *who looked just like their father* [my emphasis]” (p. 154). However, Meena is humiliated by her mother-in-law when Ram requests his parents to travel to Delhi for the cradling ceremony. The mother-in-law, in an angry tone, taunts Meena over the phone call: “What’s your problem?” she demanded. “It’s not as *if your precious wife has given birth to them. She was resting in London all this time, while some poor woman carried her burden for her* [my emphasis]” (p. 154).

The grief of the intending mothers qualifies as disenfranchised grief. The intending mothers’ guilt, depression, alienation, and the loss of the experience of birthing and carrying a child often do not receive due acknowledgement from society. They are denied the social right to mourn their loss.^52^ The intending mothers may not have been physically involved in bearing a child, but metaphorically they might be bearing the pain of reproduction for years: pregnant with the dream to bear her child, the woman in the process encounters multiple layers of societal deaths and medical interventions – sometimes experiencing the pain in her body and sometimes helplessly witnessing others taking the pain on her behalf. Surrogacy is an emotional rollercoaster. Garrod and Pascal state that “the experience of disenfranchised grief has many twists and turns. This is particularly the case in situations that have an external cause of celebrations, but in fact contains internal loss, embodied betrayal, and double jeopardy” (p. 6).^53^ The grief and plight of Meena end when clad in “new maroon Kanjeevaram silk sari with a big gold and turquoise border”, she seats in the cradling ceremony where she finally earns the prestige of being a mother (p. 153).^19^ Although a pinch of insecurity is shown by Meena because of the presence of Alice who has to accompany the couple to breastfeed the twins, finally Meena succeeds in creating her perfect (bioengineered) bionormative family. The Kanjeevaram silk sari and the gold ornaments carry symbolic connotations. They are worn by women who have the socially recognised credentials to participate in auspicious occasions. Hence, the sari and the ornament resemble Meena’s symbolic capital.

The film *Filhaal* is discussed next. Although the film is approximately two decades old, the film is revisited with a renewed attention to unfold the affective turmoil involved in altruistic surrogacy, an issue we need to discuss to reflect on the moral and ethical imperatives of altruistic surrogacy.

### Gulzar’s *Filhaal*

The film *Filhaal* (in Hindi, the word *filhaal* means “for a time being”) depicts the life of Sia, the surrogate, and dramatises the life of Rewa, a family-oriented girl who has always dreamt of a stable and happy married life. In contrast to Sia, her best friend, Rewa aspires to be a mother soon after her marriage. To fulfil her dream, Rewa marries Dhruv and they both hope to have their perfect family soon. However, their dream falls apart when Rewa faces difficulties conceiving a child. Despite her husband Dhruv’s support and suggestion to go ahead with adoption, she fails to accept the reality and goes for multiple medical consultancies to find a way to have her own baby. At one phase of her treatment, she conceives and there is a surge of happiness in the family. The family gets busy organising rituals to bless the would-be mother and to welcome the baby, but the love, attention, and care Rewa enjoys ends with a miscarriage. The episode proves to be catastrophic for Rewa as it deconstructs her narrative of reproductive futurism. Her reproductive trauma may be explained as a “generational change”, a form of hysteresis born out of the friction between Rewa’s real self and the desired self, informed by her notion of women in her family who have defined their womanhood by being a mother. Nandy^54^ states that the rituals, customs, and marriage as an institution stand on the foundation of reproductive futurism.

Similar to *Baby Makers*, in the film *Filhaal*, the guilt of not carrying and birthing the child stays with Rewa throughout the phase of the surrogacy. Sia, her best friend, agrees to be the surrogate mother, carrying Rewa and Dhruv’s baby conceived through IVF. The affective arrangement between Rewa, Dhruv, and Sia causes emotional upheavals that may be further explained by the theory of abjection. Abjection may be defined as an intensive force where a person cannot offer a definite meaning to his/her emotions. For Rewa, the journey of having a child of her own through surrogacy triggers mixed feelings. The fact that she cannot experience the pregnancy in her body sickens her, but at the same time the “certainty of [having her own baby] which she is proud of” pushes her to cling to the procedure (p. 125).^29^ The lack of the embodied experience of feeling the growth of the child in her womb produces a sense of voidness in Rewa. This experience is aptly captured in the episode where she and her husband Dhruv are dramatised enjoying an anticipatory talk about how the baby would look after the birth.

**Rewa:** During pregnancy, women love to eat tangy food items.

**Dhruv:** Then ask Sia what she feels like having.

**Rewa:** [A long silence, Rewa turns sad. Her facial expression and bodily gestures with melancholic background sound draw attention to the emptiness that glides down upon the protagonist] I heard it is possible to know the gender of the baby by looking at the stomach of the mother.

**Rewa:** It will be a baby girl. If it is a girl, a mother feels like having tangy food, and the mother’s belly bulges upward.

**Dhruv:** You should ask Sia then.

**Rewa:** [A long silence, she places her hand on her tummy and moves the hand upward and tries to experience how it feels to have a baby inside when it starts growing gradually and the stomach grows fat] *But, the baby is not in my stomach* [a long pause] (1:33:25–1:35:37). [My emphasis]

Rewa’s experience takes us back to the grief of Parvati whose loss is articulated by Ganga as well. Rewa can never experience “pregnancy milestone like feeling the baby kick” (para 3).^49^ Teman explains how the lack of “privileged embodied knowledge” of the foetus makes the intending mother feel insecure (p. 127).^2^ Rewa feels a loss of control over everything around her. She wants to take care of Sia with an intention to be with her child (1:36:36), but Sia, being a working woman, does not stay at home. Rewa gets scared. She feels that her inability to be in constant touch with Sia might affect her maternal bond with her child growing in her friend’s womb. In one of the episodes, Sia has to go to the office and does not return home (1:42:13). Anger, irritation, and sadness engulf Rewa. At this phase, Rewa receives a call from her mother:

**Mother:** How are you Rewa? What are you doing?

**Rewa:** Waiting. Waiting for Sia to return home. She has not returned and hence, there is a delay in taking her medicines.

**Mother:** [in a casual tone and a smile] *She is pregnant and not sick. She is going to become a mother soon.* [My emphasis]

**Rewa:** *Who*! *Who is becoming the mother*? [My emphasis]

**Mother:** [in a reluctant tone] Sia, she has conceived the baby and hence she is also the mother. [pause] Y*ou are a mother too*. [My emphasis]

**Rewa:** Hmm … [Gazing at the medicine for a while, Rewa goes to sleep with a feeling of learned helplessness] (1:40:16–1:41:24).

For Rewa, the word “waiting” has a literal as well as a metaphorical connotation. She is waiting for her friend to return home, but at the same time, there is a long wait within her to feel her baby in her lap. Her mother’s explanation of motherhood further accentuates her grief and confusion. The sociocultural notion of motherhood comes in conflict with the medico-legal definition of motherhood. Biologically, the child in Sia’s womb is Rewa’s child but at the same time, the embodied feeling of having a child within her is missing. The capsules prescribed by the doctor should be taken by Sia, not by Rewa. As the biological mother of the child, Rewa is available to take the medicine; however, the medicine will have no significance in her body as she does not embody the child. Her womb is empty and hence Rewa starts questioning herself as to what extent she should be acknowledged as the mother of her child. Gradually Rewa develops anger and disgust towards Sia. She starts abhorring Sia’s presence. In one of the scenes, when Rewa requests her husband to take her out for dinner, Dhruv immediately books a table and phones Sia to join them for dinner. When asked the reason for inviting Sia as well, Dhruv says “I want to spend time with our baby as well, so Sia has to be there” (1:46:37–1:47:21).

The integral presence of Sia is smeared with a feeling of learned helplessness as well as abjection for Rewa: the birthing mother’s presence is welcoming as well as not desired. As explained by Kristeva, it is a state where we look at someone with revulsion. The presence of Sia has a liminal connotation for Rewa. Sia is the gestational surrogate of her child whom she loves dearly, but at the same time, her best friend’s presence reminds Rewa of what she is lacking within her. Who is Sia? One of the mothers of her baby, the friend, or the woman whose womb she has taken for *Filhaal*. Rewa experiences an epistemic clash and fails to locate Sia’s identity in binaries. Rewa experiences an order of abjection where Sia is transformed into a liminal being who is “quite close” to Rewa but at the same time, the intending mother abhors the thought of assimilating the surrogate mother into her life. Sia occupies a non-presence state in Rewa’s life. Rewa fails to recognise her kinship with Sia, which is explained by Kristeva as an affect where “the liminal and unaccommodated being trigger a feeling of horror and disgust because they foreground the fragility of binaries and epistemic purity of symbolic order that inform our normative and dichotomous way of constructing meaning of sociocultural phenomenon with which we interact and are engaged in our everyday life” (pp. 6–7).^55^

In *Baby Makers*, Meena and Sharada restore their habitus with the arrival of their babies. In contrast to them, Rewa’s struggle in the film *Filhaal* seems to be endless. Rewa plunges into grief and insecurity and in the heat of the moment demands Sia to “abort the baby” (2:11:32). Her husband and Sia look at Rewa with fear and disgust. The words fill Rewa with disgust too, and in rage, she finally leaves her husband’s house. This behaviour does not mean that intending mothers, who in a rage take an instant decision, are monstrous mothers. As discussed, a person’s affective disposition – personal relations, bodily abilities, traumatic experiences – has a strong impact on his/her bodily and mental activities. Sociologist Stuvoy appeals to move away from the notion of reproductive assistance as a substitution or transaction. She urges practising the notion of surrogacy as a “relational being-together” (p. 39).^3^ *Baby Makers* and *Filhaal* dramatise the difficulty of achieving an affective relationship in this situation where the presence of the surrogate mothers may trigger anxiety and abjection in the intending mothers. This situation arises because of the abjection that technologically driven reproductive relationality brings into being. However, the fictional narratives do not deny the possibilities for developing affective relationalities. Rewa undergoes an inner tussle with her inhibitions and emotional challenges and when she learns that her friend has been admitted to hospital and in a critical state, she reaches the hospital immediately. In the film *Filhaal*, in the final scene Rewa says that her best friend Sia is not “*filhaal ki Maa*” (mother for the time being) but she is also one of the mothers of her baby (2:25:28).

## Conclusion

Through a close analysis of the works of fiction such as *Kartikeya* and *Baby Makers* and the film *Filhaal*, this article underscores how the decision-making around surrogacy may turn into a complex affective journey that could cause the intending mother to experience depression, anxiety, and alienation. By foregrounding the significance of the selected fictional narratives and emphasising the need to acknowledge the grief of the intending mother, this study contributes to research narratives on SRHR. As shown many times in the fictional narratives, the pain and grief of the intending mothers are not acknowledged by the family members, and their decision to use the surrogacy option is interpreted as an easy recourse. Analysis of the narratives of the selected texts demonstrates how these negative attitudes harm the reproductive decision-making of the mothers, pushing them to experience further trauma and discrimination. Society’s prejudiced and biased perspectives towards the decision-making and motherhood of intending parents are an assault on women’s human rights. We should recognise the fact that reproductive trauma is an emotional scar, an affective disposition that gradually consumes the well-being of the parents. Fictional narratives such as *Kartikeya, Baby Makers,* and *Filhaal* highlight the bereaved and traumatised subjectivity of the intending mothers, strongly refuting the dominant portrayal of women deciding to avail surrogacy for baby making as heartless capitalist monsters.

This study concludes by stating that we need to take the discussion on reproductive trauma, vulnerable mothers, and disenfranchised grief forward by strongly recommending counselling during and post surrogacy procedures to help the couple, more specifically the intending mother, develop emotional resilience to celebrate the journey of motherhood, which has been realised through a complex process of biotechnological intervention. Intergenerational counselling would help family members to understand and support the intending mother’s reproductive decision-making. Sexuality education should enable youths to understand the integral connectivity between human rights and women’s SRHR. Informing students about the different orders of human reproduction that are available will offer them new ways of interpreting normative notions of motherhood, parenting, and family-making. People working in media should collaborate with academics, social workers, and fertility experts to understand the complex affective issues of surrogacy practices. Efforts should be made by the media to produce counter-narratives challenging and breaking the stereotypical images of intending mothers as depicted in mainstream society.
